# Strategies and distinguishing characteristics of faculty change agents teaching public health: a study on innovative teaching in higher education

**DOI:** 10.3389/fpubh.2026.1694800

**Published:** 2026-03-18

**Authors:** Elizabeth M. Weist, Elizabeth Armstrong-Mensah, Sarah E. Cprek, Dabney P. Evans, Jonathan Garcia, Sophie Godley, Jessica S. Kruger, Leah Christina Neubauer, Elizabeth Reisinger Walker, Juan S. Leon

**Affiliations:** 1 Association of Schools and Programs of Public Health, Washington, DC, United States; 2 Department of Health Policy and Behavioral Sciences, School of Public Health, Georgia State University, Atlanta, GA, United States; 3 College of Public Health, University of Kentucky, Lexington, KY, United States; 4 Hubert Department of Global Health, Rollins School of Public Health, Emory University, Atlanta, GA, United States; 5 College of Health, Oregon State University, Corvallis, OR, United States; 6 Department of Community Health Sciences, School of Public Health, Boston University, Boston, MA, United States; 7 School of Public Health and Health Professions, University at Buffalo, Buffalo, NY, United States; 8 Department of Health Behavior and Health Equity, School of Public Health, University of Michigan, Ann Arbor, MI, United States; 9 Department of Behavioral, Social, and Health Education Sciences, Rollins School of Public Health, Emory University, Atlanta, GA, United States

**Keywords:** academic public health, curricular transformation, DEIJ, higher education, pedagogy, scholarship of teaching and learning, student learning outcomes, teaching excellence

## Abstract

This study investigates the strategies and distinguishing characteristics of 12 Association of Schools and Programs of Public Health (ASPPH) Early Career Teaching Excellence Award winners, analyzing their alignment with ASPPH’s *Framing the Future 2030* (FTF 2030) and the Robert Wood Johnson Foundation’s *Transforming Academia for Equity* (TAE) rubric. Both frameworks call for inclusive excellence, transformative teaching, and strong community partnerships to prepare a public health workforce equipped to meet evolving challenges. The study situates these educators as change agents operating within a shifting higher education and public health landscape marked by a lack of support and incentives for teaching within a challenging public health climate. Qualitative, exploratory methods engaged awardees through one in-person focus group and multiple individual virtual interviews. These conversations explored strategies aligned with both the FTF 2030 and TAE frameworks. The researchers analyzed the data using a modified grounded theory approach and mapped findings to both frameworks, validating themes through member-checking with participating coauthors. Three cross-cutting findings emerged as distinguishing characteristics of the awardees: (1) adoption of transformative teaching approaches; (2) teaching as a vocational calling; and (3) advancing change beyond the classroom. Quantitative findings showed that awardees implemented 50 strategies aligned with FTF 2030 categories of practice and 18 with TAE categories of impact, emphasizing student-centered learning, institutional change, and community-engaged practice. Facilitators included supportive leadership and teaching-focused learning communities. Barriers included undervaluing teaching relative to research and the overreliance on student evaluations. Results highlight how these exemplary educators deploy a variety of innovative strategies and practices to prepare students for the workforce, foster belonging, and drive institutional change to promote teaching excellence. Their work underscores the importance of empathy, humility, and resilience as core professional competencies. Sustaining and scaling such innovation requires institutionalized support and resources that recognize teaching as central to academic public health’s mission. In times of disruption, these transformative educators serve as inspiration to their peers and enhance their institutions’ reputation for teaching excellence, thus increasing prospective students’ interest as well as better preparing graduates for professional practice in protecting the public’s health during the current and future challenging times.

## 1 Introduction

Public health professionals at governmental public health agencies, within academia, and in practice settings have driven numerous public health successes over the past century through quiet and steady evidence-based research, teaching, and application in communities (1), often using “behind the scenes” efforts. These achievements are even more notable today given the current challenges facing the public health workforce (2).

A 25-year retrospective systematic review, commissioned by the Robert Wood Johnson Foundation (RWJF), identified the urgent need to: develop a more diverse public health workforce to match the changing demographics of the United States; recruit and retain public health professionals to fill the gap left by an aging workforce; and increase education and training in public health to address the major workforce shortage (3). Recently the public health workforce has experienced mounting pressures, arising from demands of the coronavirus disease 2019 (COVID-19) weak pandemic response (4), and rising vaccine hesitancy (5, 6), leading to disease outbreaks such as measles (7). The proliferation of misinformation and disinformation (8, 9) along with the growing public distrust in science (10–12) confronting public health leaders has challenged the public’s ability to both understand and trust the profession (13).

As challenges to public health grow and evolve, it is imperative to adapt strategies that serve the dual goals of “improving the quality of our teaching and enhancing student learning outcomes” (14) to build future members of the public health workforce (13, 15–17). In addition to graduate education in public health, baccalaureate education in public health is increasingly available to undergraduate students in two unique frameworks, either as part of a general liberal arts education or within pre-professional practice-based programs (18). While academic institutions at all levels prepare learners for public health careers, it is unclear whether current educational strategies are keeping pace (19, 20). Students for example are seeking educational approaches that better prepare them for upstream improvements to the health system that will impact public health (21) and larger questions remain about the extent to which higher education meets the needs of the moment, including its contribution to income segregation and intergenerational mobility (22), accountability to the public (23), and outcomes in delivering a return on investment (24).

In response to these challenges and opportunities, as well as to calls to action for increasing teaching excellence in public health (25–30), the Association of Schools and Programs of Public Health (ASPPH) set forth in 2020 in “Framing the Future 2030: Education for Public Health” (FTF 2030) a “vision” for “equitable, quality education in public health…for everyone everywhere.” (13). ASPPH is a global association representing accredited academic public health institutions (31).

**Figure 1:**
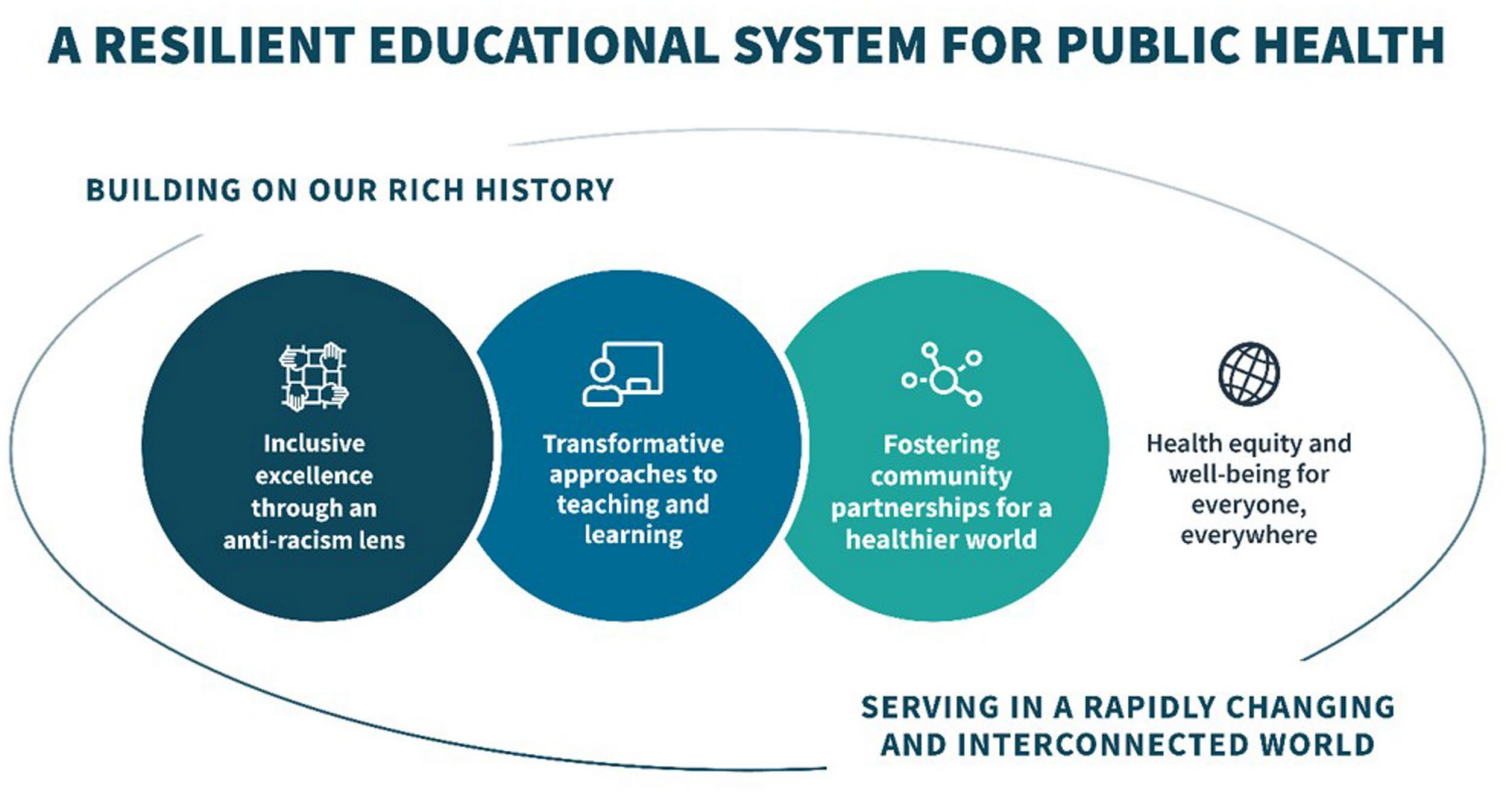
ASPPH vision for framing the future 2030 (35).

From 2020–2024, ASPPH created and charged three FTF 2030 expert panels, composed of members from ASPPH institutions and partner organizations, to focus on three key objectives to guide institutional missions for improving teaching for public health: (1) advancing excellence and equity; (2) modernizing education; and (3) deepening engagement. These panels delivered three reports: (1) *Building Inclusive Excellence Through an Anti-Racism Lens: Transformative Action in Academic Public Health* (32); *Transformative Approaches to Teaching and Learning Report* (33); and *Fostering Community Partnerships for a Healthier World: A Call to Action and Framework* (34), and an executive summary (35) in March 2024. These three reports included twelve recommendations comprising ASPPH’s call to action (Table 1). FTF 2030 supports ASPPH’s overall strategy to build a resilient educational system for public health that serves everyone, everywhere (Figure 1).

**Table 1:** ASPPH framing the future 2030 call to action, 12 recommendations (87)*

Advancing excellence & equity	Modernizing education	Deepening engagement
*Create and support* inclusive and anti-racist teaching, learning, and working environments by articulating norms and values, training and supporting constituents, and building infrastructures and systems to bolster efforts. [IE-1]	*Center* civic engagement, cross-sectoral collaboration, and community partnerships as essential elements of the learning experience. [TA-6]	*Position* academic public health in partnership with communities. [FCP-10]
*Establish* necessary partnerships and secure resources, including leadership commitments, to sustain efforts and assure accountability. [IE-2]	*Ground* education in collective action to assess and address the social determinants of health. [TA-7]	*Deploy* strategies to support and sustain successful partnerships. [FCP-11]
*Increase* commitment, perseverance, discipline, and consistency of inclusive efforts among all members of our respective institutions. [IE-3]	*Use* active learning and support lifelong learning to prepare diverse, practice-ready professionals. [TA-8]	*Develop* curricula to prepare learners with knowledge, skills, and mindsets for more effective partnering. [FCP-12]
*Develop* system-wide initiatives to promote and assure accountability for inclusive excellence through an anti-racism lens. [IE-4]	*Assure* ongoing training in evidence-based frameworks, methods, and technologies for teaching, learning, and assessment of educational outcomes. [TA-9]	
*Share* initiatives aimed at inclusive excellence through an anti-racism lens to accelerate institutionalization and integration across academic public health. [IE-5]		

* Text in brackets “[abc…]” represents each of the three FTF 2030 themes, with “Inclusive Excellence…” abbreviated to “IE,” “Transformative Approaches…” shorted to “TA,” and “Fostering Community Partnerships…” abbreviated to “FCP” which is followed by the number of the recommendation within each theme.

As a complement to FTF 2030, ASPPH is funded to advance the dissemination of the Robert Wood Johnson Foundation Transforming Academia for Equity (TAE) pilot project (36). The TAE project that inspired the title for this journal’s special issue builds on ASPPH’s commitment to dismantling racism and promoting inclusive excellence in academic public health. More specifically, ASPPH’s *Dismantling Racism and Structural Racism in Academic Public Health: A Framework Report* (37) undergirds its efforts to advance TAE desired outcomes and serves as a foundation for the FTF 2030 “Inclusive Excellence through an Anti-Racism Lens” (Figure 1) expert panel’s deliberations and ensuing work (32). TAE and FTF 2030 leaders are natural partners in seeking to identify and address the root causes of health inequities in the US. Both efforts serve public health via academic leaders, faculty, staff, students, and partners who collaborate to leverage commitments and resources in support of educators who engage in high-quality teaching that prepares students for practice in a complex and pluralistic world.

**Figure 2:**
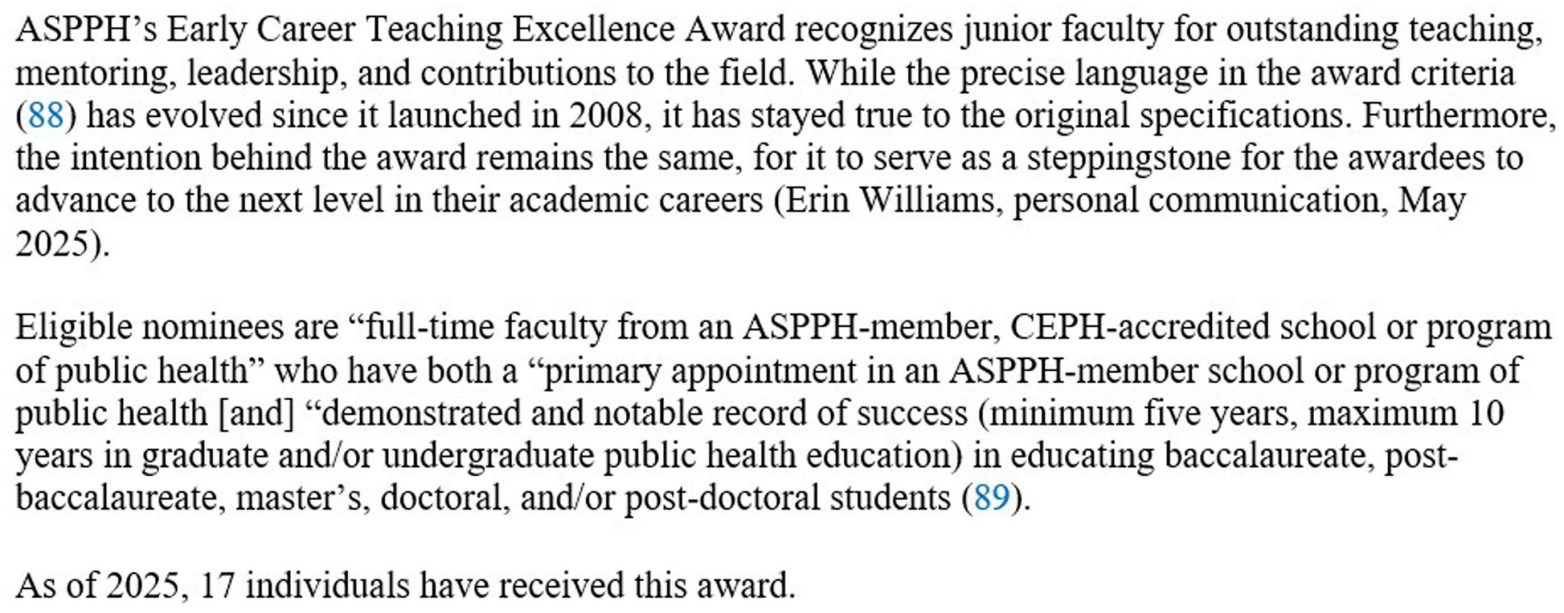
About the ASPPH early career teaching excellence award.

To support educators in public health who are striving to teach effectively while facing unprecedented challenges, the goal of this study is to identify the characteristics of innovative educators in public health and their strategies for effective teaching during the turbulence in academic public health. The researchers chose ASPPH’s cohort of Early Career Teaching Excellence Award winners (termed “awardees”, see Figure 2 for criteria and eligibility for the award) as a representative pool of effective educators.

Two key questions underlie our exploration of the characteristics of effective educators and their strategies aligned with the FTF 2030 and TAE objectives: (1) what is fundamental and important in educators’ roles (individually) and in schools and programs of public health (institutionally) in preparing graduates for successful practice in today’s complex higher education and health ecosystem?; and (2) how do educators function within an academic culture with few incentives for transformative teaching, especially amidst financial and political headwinds facing institutions today? ASPPH defines transformative teaching as follows: “Transformative education involves critical exploration, questioning assumptions, and is achieved through teaching and learning that engages and empowers learners. The goal of transformative education in public health is to prepare learners to make informed decisions and drive meaningful actions, both locally and globally, at individual, institutional, and community levels” (32).

## 2 Materials and methods

### 2.1 Study design and rationale

This study employed an exploratory approach using both qualitative and quantitative methods with a cohort of recognized award-winning educators, ASPPH’s Early Career Teaching Excellence awardees. The supposition was that these exemplars exhibit effective teaching practices. The study focuses on awardees’ deidentified and aggregated experiences in the academic public health mission—research, teaching, and practice—with a focus on their education/teaching role.

### 2.2 Participant identification and selection

In January 2025, the lead researcher (EMW) emailed invitations to all 17 recipients of the ASPPH Early Career Teaching Excellence Award to participate. Those who accepted (*n* = 12) signed a consent document “Terms of Participation,” filled out a demographic poll, and opted to join either an in-person focus group discussion or an individual virtual interview (jointly termed “conversations”). Conversations took place between March–June 2025.

### 2.3 Ethical considerations

Emory University’s Institutional Review Board determined that this study did not require formal institutional ethical review because it was not considered “research” in the federal regulations. Consent to participate in the study was solicited through the entire data collection process. No compensation was offered and there were no incentives that could unduly influence participation. Researchers followed all ethical standards for social and educational research, including those outlined in the Declaration of Helsinki, reviewing study protocols to ensure participant autonomy, confidentiality, and data security.

### 2.4 Data collection procedures

Awardees were asked about their efforts and perspectives related to three Framing the Future (FTF) 2030 objectives: (1) building inclusive excellence through an anti-racism lens, (2) transformative approaches to teaching and learning and, (3) fostering community partnerships for a healthier world, described in three expert panel reports (32–34, Table 1) as well as their efforts and perspectives related to ASPPH’s “Transforming Academia for Equity (TAE) Desired Outcomes Rubric” (Supplementary Table 1). This rubric included: (1) inclusive leadership and representation that influence institutional change, (2) engagement in institutional learning and transformation that leads or substantially contributes to institutional diversity, equity, and inclusion (DEI) efforts, and (3) demonstration of clear and measurable impacts on attitudes, awareness, and behavior related to DEI. Each desired outcome is associated with six sample measurable indicators. Data collection focused on how awardees implement objectives from both frameworks in their educational practice.

Each participant was assigned to one of two facilitator-guided conversation(s) formats to collect data. The first format consisted of a single in-person focus group with three awardees, scheduled for two hours; the guided discussion component lasted approximately 70 min. The second format involved one-on-one virtual conversations conducted via Zoom with nine separate awardees. These sessions were scheduled for 60 min each, with actual interview lengths ranging from 35 to 55 min. Following principles of community engaged research (38), participating awardees were invited to join the lead researcher as coauthors in this article, contributing to data collection, analysis, and writing, of which nine accepted.

### 2.5 Conversation protocol and facilitation

First, the facilitator greeted the participants and introduced the purpose of the meeting. Participants and the facilitator reviewed the “Terms of Participation” and participants offered verbal affirmation and agreement to sign the document. The in-person focus group was recorded from a nearby laptop running Zoom’s built-in audio recording functionality. The virtual conversations were recorded using Zoom’s built-in audio and video recording functionality. Conversations followed a semi-structured interview protocol consisting of seven open-ended guiding questions (Table 2).

**Table 2:** Seven open-ended questions for guiding the awardee conversations.

Question No.	Questions
1.	Since winning the Teaching Award [for the 2025 winner only: Since launching your teaching career…], please share one example of how your teaching methods and strategies have grown and evolved?
2.	Talk about your efforts to transform your unit’s space for opportunity, engagement, and excellence in research, teaching, and practice.
3.	What is the most important facilitator for your teaching practice that manifests opportunity, engagement, and excellence in one or more of the three FTF 2030 themes for: Building inclusive excellence through an anti-racism lens Innovating teaching and learning Shifting academic mindsets for improving community partnering?
4.	What is the biggest obstacle you have encountered in your teaching practice in striving to manifest opportunity, engagement, and excellence in one or more of the three FTF 2030 themes for: Building inclusive excellence through an anti-racism lens Innovating teaching and learning Shifting academic mindsets for improving community partnering?
5.	Share a teaching method or process that resulted *measurably* in improving your students’ ability to address the health of all populations, in particular the most at-risk and/or vulnerable in our society.
6.	What lessons have you learned about integrating technology into your teaching and student learning?
7.	What advice would you give to an early career public health teacher for achieving teaching excellence for serving all students, in particular the ones with the greatest needs and opportunities for growth?

### 2.6 Analytical framework

The research team conducted qualitative and quantitative data analyses. The qualitative data analysis followed a community-engaged, modified grounded theory approach (39–41) in which the lead researcher pattern coded the findings to identify the awardees’ common shared characteristics. For the quantitative phase of the study, the lead researcher developed an initial set of *a priori* codes based on the 12 FTF 2030 recommendations (Table 1) and the TAE rubric with its three desired outcomes and six “Sample Measurable Indicators” (Supplementary Table 1).

### 2.7 Coding strategy and analysis

Two phases of coding were conducted to identify: (1) common characteristics of the 12 awardees, and (2) awardees’ strategies aligned with FTF 2030 and TAE.

The first phase of analysis was qualitative and focused on identifying common salient characteristics of the 12 awardees. Following completion of the first six conversations (the in-person focus group of three awardees and 3 virtual one-on-one conversations), the lead researcher downloaded and reviewed the Zoom-generated transcripts. Transcripts were deidentified and edited for clarity and brevity, with minor reorganization of content to align responses with the relevant guiding questions. Each transcript was anonymized using a numerical identifier (01–06), and each was read completely a minimum of two times, with two additional rounds of refining the transcripts for clarity and brevity to ensure coherence and consistency.

The lead researcher then engaged in open coding, manually highlighting potentially salient responses to identify key concepts. Open coding served as an initial pass to surface meaningful content from the data. This was followed by manual *in vivo* coding, in which participants’ exact words were used to generate codes, a method that facilitates the development of themes grounded in participant language (41). Through iterative analysis, the lead researcher identified seven preliminary themes using axial coding, which enabled the exploration of relationships among themes and subthemes. Excerpts were organized under the corresponding themes using a constant comparative method to assess consistency and thematic saturation. This process led to the emergence of an eighth theme. The lead researcher subsequently revisited earlier transcripts to identify additional supporting evidence for this new theme and the seven other themes. The remaining six transcripts were processed using the same analytical framework. For comparative purposes, responses were also grouped according to the seven open-ended questions guiding the conversations (Table 2), allowing for thematic analysis within and across the questions.

After a final, comprehensive review of the eight themes with selected excerpts affirmed as representative of each theme, the lead researcher prompted ChatGPT (42) to synthesize the data into salient themes (43). Upon receipt of the AI results, the lead researcher checked the artificial intelligence product, finding the AI product both deeper and more generic than her findings. The lead researcher adopted some suggestions where there was missing content in her work and discarded many others as not only distracting to the analysis but not fitting with the evidence grounded in conversations with the awardees as supported by excerpts. She wrote memos on the eight themes with evidence of the awardees’ distinguishing features and characteristics drawn from direct excerpts.

The second phase of analysis was quantitative and focused on identifying awardee strategies that aligned with the FTF 2030 recommendations and the TAE rubric. All twelve transcripts were systematically re-examined using deductive coding to identify and extract excerpts that demonstrated fulfillment of the frameworks. The lead researcher coded the data against the twelve FTF 2030 recommendations (Table 1) and, separately, against the three desired outcomes of the TAE rubric and six associated indicators for each outcome (Supplementary Table 1).

### 2.8 Member engagement and co-interpretation

At the data analysis and writing stage, the lead researcher called upon the awardees who had agreed to engage in co-creating and writing up the study findings. These nine coauthors reviewed initial themes with the lead researcher and collaborated in analyzing the findings in dialog with existing literature. Member-checking (44) took place in an open virtual meeting with the coauthors to discuss the first draft of the manuscript, review major themes, and elicit feedback. Additional themes (e.g., the complexity of resilience across institutional contexts) emerged in dynamic conversation as participants reflected on findings through the process of developing, refining, and submitting the final draft of the manuscript.

## 3 Results

### 3.1 Awardee demographics

To describe the awardees’ demographics, the researchers analyzed responses to a participant poll (Table 3) that showed the twelve awardees have worked a range of 8–26 years in higher education and have advanced in rank since winning the award, with 25% currently as Professor (0% at time of the award) and 67% as Associate Professor (8% at the time of the award). The majority (58%) are in the 45–54 year-old age bracket, identify as women (58%), non-Hispanic (83%), and white (82%).

**Table 3:** Participating awardees’ responses to the demographic poll.

Demographic variable (*n* = 12)	Value*
Years worked in higher education	8–26 years (Median: 15.5)
Current academic rank	
Professor	25% (3)
Associate professor	67% (8)
Other†	8% (1)
Academic rank at time of award	
Professor	0% (0)
Associate professor	8% (1)
Assistant professor	67% (8)
Other‡	25% (3)
Current age range	
35–44 years old	17% (2)
45–54 years old	58% (7)
55–64 years old	25% (3)
Identifies as woman	58% (7)
Hispanic or Latino	17% (2)
Race (multiple selections allowed *n* = 11)	
American Indian or Alaska Native	9% (1)
Asian	9% (1)
Black or African American	9% (1)
White	82% (9)

*Unless indicated, number in parentheses represent counts.

†Other: Clinical associate professor.

‡Other: Assistant scientist, clinical associate professor, teaching associate professor.

### 3.2 Awardee strategy category findings

To understand the awardee strategies that implemented the two frameworks [Framing the Future (FTF) and Transforming Academia for Equity (TAE)], the lead researcher used induction to identify six FTF 2030 strategy categories (Table 4) and four TAE strategy categories (Table 5). These qualitative findings were grounded in the awardee conversations and illustrated by quoted excerpts.

**Table 4:** Findings organized by FTF 2030-aligned strategy category, example awardee strategies, and illustrative quotes.

Strategy category	Example awardee strategies	Illustrative quotes
A. Prepare students for community-engaged practice	Establish community partnerships with the department of health (DOH) and cross-sectoral collaboration with the K–12 system Align capstones with local DOH accreditation Create a community of practice among academic and non-academic partners	“I want students to learn…with me and with their peers, not on the backs of the community.”
B. Use student-centered practices	Co-create norms with students to increase their active engagement Engage non-native English speakers in class discussions (with intention and in a supportive manner) Pivot to address critical issues of the day, which may require setting aside the lesson plan	“See the assets in your students.”
C. Deploy strategic systemic levers of change	Roll-out, evaluate, and disseminate a DEI reflection tool Accreditation and visioning for the future of the school Build a diverse leadership team (e.g., senior administrators, faculty leaders, and students) to coordinate institutional DEI efforts across academic and community sectors (aka “container approach”)	“I was also involved deeply in helping with accreditation of our school and reimagining what our school would be like.”
D. Apply pedagogical techniques	Engage in self-critique to improve anti-racism teaching and learning Use case-studies [e.g., Harvard’s Global Health Delivery Case Collection and Transparency in Learning and Teaching (TILT) strategies] Use cognitive dissonance in guiding students to step out of comfort zones	“I tell my students: ‘This is the way that I’m scaffolding the learning experience. These are the pedagogical principles that I’m using.’”
E. Lead in faculty development	Outreach to others to elevate their teaching and assessments Volunteer counseling work at the university’s teaching center Lead seminars and orientation workshops on teaching strategies	“I gave a lot of talks about teaching, learning how to become a mindful educator, and to elevate teaching in our school.”
F. Implement extracurricular strategies	Offer a listening session with marginalized students to increase the sense of belonging Include students in the implementation of a speakers’ series Lead with racism and anti-racism exploration in community settings	“I often share examples with students of mistakes in community [work]… I want the classroom to be a place where there’s an opportunity to learn and grow and be embarrassed, and to learn to cope with that.”

**Table 5:** Findings organized by TAE-aligned strategy category, example awardee strategies, and illustrative quotes.

Strategy category	Example awardee strategies	Illustrative quotes
A. Execute institutional change strategies	Change promotion and tenure guidelines to further recognize teaching excellence Implement a community of practice with an associated learning tool Co-create a departmental DEI committee	“[W]e have made impacts in promotion and tenure guidelines…”
B. Implement new equity content and curricula	Initiate a new core curriculum that includes equity content, for the undergraduate program Institute a Health Equity course Build a study abroad program that exposes students to diverse perspectives and others lived experiences	“We are developing a new curriculum for the core education for the undergraduate program, and several people are spearheading that.”
C. Use student-centered strategies	Execute a successful group peer-advising model Partner with students in creating a new justice, equity, diversity, and inclusion hub that increases a sense of belonging Use student feedback to inform equity-based changes in the curriculum	“We developed a model of group advising where we would have two faculty advisors for a group of 8 to 10 students…”
D. Advance student growth in equity-oriented learning	Measure the impact of structural bias and cultural humility learning using the CEPH competencies Increase in student understanding about research harm in communities from studying “The Immortal Life of Henrietta Lacks”	“[I]n my qualitative methods class I teach and assess CEPH’s foundational competencies related to structural racism and cultural humility…”

#### 3.2.1 Phase 1 findings organized by FTF 2030-aligned strategy category

Six strategy categories, lettered A-F, described the 50 FTF 2030-related findings, as explained below and depicted in Table 4.

- “Prepare students for community-engaged practice” was exemplified by setting up structures for engagement with community partners (e.g., building relationships with local health departments and collaborating cross-sectorally with the K-12 system), aligning the learning experience in response to real-world needs (e.g., establishing capstone experiences that serve department of health accreditation purposes), and ensuring students and community partners are prepared to interact with each other (e.g., establishing a community of practice among both academic and non-academic partners).
- “Use student-centered strategies” was exemplified by practices in which educators engage with their students, drawing them out from the start by establishing ground rules for the classroom (e.g., co-creating norms to increase student engagement), ensuring that all students participate in class discussions (e.g., engaging non-native English speakers, with intention and support), and responding to watershed moments in current events (e.g., pivoting to address critical issues of the day, even if it requires setting aside the lesson plan).
- “Deploy strategic systemic levers of change” was exemplified by awardee participation outside the classroom, such as by creating cross-departmental evaluation tools (e.g., development of a DEI reflection tool that led to curricular changes), participating in school-wide planning initiatives (e.g., accreditation and visioning with colleagues and leaders), and aligning players across the university to integrate efforts (e.g., using a “container approach,” the intentional creation of a microcosm of individuals drawn from a wide array of diverse identities, hierarchies, and roles within the academic system and partnering communities to mobilize collective engagement for implementing systemic change) (45).
- “Apply pedagogical techniques” was exemplified by the awardees’ use of an array of intentional evidence-based methods, including self-reflection (e.g., self-critiques that improve inclusive anti-racist teaching and learning, in particular), application of high-impact practices (e.g., case studies and transparency about teaching methods), and the use of cognitive dissonance and posing of queries that challenge received knowledge (e.g., encouraging students to step out of comfort zones).
- “Lead in faculty development” was exemplified by meta-activities the awardees use to support fellow faculty in their growth, including initiating faculty conversations about teaching (e.g., delivering teaching talks), mentoring faculty in teaching (e.g., volunteering counseling work at the university’s teaching center), and training peers to improve their teaching (e.g., creating faculty workshops that include teaching strategies).
- “Implement extracurricular strategies” was exemplified by offering non-classroom opportunities for students’ and partners’ learning and growth, including facilitating group sharing (e.g., offering a listening session with marginalized students to increase the sense of belonging), inviting students to lead in a public-facing event (e.g., including students as leaders in launching a speakers’ series), and bringing culturally sensitive issues to the fore with partners (e.g., leading with race and anti-racism exploration in community settings).

#### 3.2.2 Phase 1 findings organized by TAE-aligned strategy category

Four strategy categories, lettered A-D, described the 18 TAE-related findings, as explained below and depicted in Table 5.

- “Execute institutional change strategies” was exemplified by convening mission-aligned groups and exerting leadership to change the institution, including policy shifts (e.g., changing promotion and tenure guidelines), creating new institutional organizations and buttressing its efforts with resources (e.g., implementing a community of practice with and associated learning tool), and collaborating to establish a new entity (e.g., co-creating a new departmental DEI committee). This TAE strategy category overlapped with findings in the FTF 2030 category “Deploy strategic systemic levers of change” (Table 4).
- “Implement new equity content and curricula” was exemplified by the development of content and curricular structures that respond to students’ social justice and equity interests and that engage students on DEI issues, including launching new curricula (e.g., initiating a new core curriculum, inclusive of equity content, for the undergraduate program), starting a new course of study (e.g., instituting a Health Equity course), and offering new learning options (e.g., building a study abroad program that exposes students to diverse perspectives and others’ lived experiences).
- “Use student-centered strategies” was exemplified by methods to meet the students “where they are,” raise up student leaders, and respond to student feedback regarding their needs and preferences, including pilot testing and adopting a new approach to advising (e.g., executing a successful peer advising model), partnering with students to develop a stronger culture of belonging (e.g., engaging students in creating a new justice, equity, diversity, and inclusion hub that increases a sense of belonging), and eliciting student input to inform curricular changes (e.g., using student feedback to inform equity-based changes in the curriculum). This TAE strategy category overlapped with FTF 2030 findings in the strategy category of the same name “Use student-centered strategies” (Table 4).
- “Advance student growth in equity-oriented learning” was exemplified through the use of teaching strategies that document pedagogical equity objectives are met (e.g., assuring student fulfillment of CEPH’s foundational competencies related to structural racism and cultural humility) and observing the evolution of students’ grasp of equitable research methods over the years (e.g., noting of an increase in student understanding about research harm in communities from studying “The Immortal Life of Henrietta Lacks”).

#### 3.2.3 Phase 2 findings showing relationship of awardee strategies to FTF 2030 and TAE frameworks

We were interested in assessing the relationships of these strategy categories (Tables 4, 5) with the FTF 2030 recommendations (Table 1) and elements of the TAE desired outcomes rubric (Supplementary Table 1). To do so, we mapped each awardee strategy to all applicable FTF 2030 recommendations and TAE desired outcome rubric elements, then summed the number of recommendations and rubric elements operationalized within each strategy category into two new tables (Tables 6, 7).

**Table 6:** Awardee strategies operationalizing FTF 2030 recommendations.

		Strategies operationalizing FTF 2030 recommendations*		
Strategy category	Number of awardee strategies	Inclusive excellence	Transformative approaches	Fostering community partnerships
A. Prepare students for community-engaged practice	12	0	7	12
B. Use student-centered practices	11	11	1	1
C. Deploy strategic systemic levers of change	11	7	6	2
D. Apply pedagogical techniques	9	6	3	2
E. Lead in faculty development	4	0	4	0
F. Implement extracurricular strategies	3	2	1	0
Total	50	26	22	17

* Some awardee strategies fulfill more than one FTF 2030 recommendation, however, each is counted only once, within the recommendation it fits best.

**Table 7:** Awardee strategies operationalizing TAE desired outcomes.

		Strategies operationalizing TAE desired outcomes*		
Strategy category	Number of awardee strategies	Inclusive leadership and representation	Engagement in institutional learning and transformation	Impact on attitudes, awareness, and behavior
A. Execute institutional change strategies	7	4	2	2
B. Implement new equity content and curricula	5	0	5	0
C. Use student-centered strategies	4	3	1	1
D. Advance student growth in equity-oriented learning	2	0	0	2
Total	18	7	8	5

* Some excerpted strategies fulfill more than one TAE desired outcome, however, each is counted only once, within the desired outcome it fits best.

This process surfaced fifty awardee strategies that operationalize FTF 2030 recommendations (Table 6). “Prepare students for community-engaged practice” had the most (*n* = 12/50, 24%) awardee strategies and “Implement extracurricular strategies” had the least (*n* = 3/50, 6%). Among the three sets of FTF 2030 recommendations, the “Inclusive Excellence through an Anti-racism Lens” (“Inclusive Excellence” for short) objective captured the preponderance of strategies, with 52% or 26 total and five of these operationalized in one or more of the other objectives (data not shown). Most of the strategies in the “Inclusive Excellence” objective related to “inclusive” teaching and learning strategies with few strategies aligned specifically with ‘anti-racist’ teaching and learning strategies. The “Transformative Approaches to Teaching and Learning” (“Transformative Approaches” for short) objective was next with 44% or 22 strategies and with ten of these items operationalized in one or more of the other objectives (data not shown). Lastly, the “Fostering Community Partnerships for a Healthier World” (“Fostering Community Partnerships” for short) objective garnered 34% or 17 of the strategies and with 12 of these items operationalized in one or more of the other objectives (data not shown), revealing interesting intersections particularly between the “Transformative Approaches” and “Fostering Community Partnerships” objectives. See these 50 FTF 2030 strategies coded into six categories of practice, lettered A-F (Supplementary Table 2).

Eighteen awardee strategies operationalize elements of the TAE desired outcomes rubric (Table 7). “Execute institutional change strategies” was cited the most (7/18, 39%) and “Advance student growth in equity-oriented learning” the least (2/18, 11%). Further, coding against the rubric, shows that 39% (*n* = 7/18) of the awardee strategies support the first TAE desired outcome “Inclusive Leadership and Representation.” Of these, 71% (*n* = 5/7) were in the lower spectrum indicators, namely the level 1 indicator “Leadership Roles,” such as “DEI-focused committees, faculty workshops,” and the level 2 indicator “Initiatives Led,” such as “Programs that support marginalized groups, mentoring programs” (data not shown). A slightly higher percentage, 44% (*n* = 8/18) of the examples support the second TAE desired outcome “Engagement in Institutional Learning and Transformation.” Of these, 50% (*n* = 4/8) of these examples reside in the lower level 2 indicator “Curriculum Work,” such as “Development/redesign of syllabi/courses to include inclusive content” indicator, an activity that is deeply embedded already within the core teaching work of a university (data not shown). Lastly, 28% (*n* = 5/18) of the examples support the third TAE desired outcome “Impact on Awareness, Attitudes, and Behavior.” Of these, 60% (*n* = 3/5) of the strategies reside in the lower two rungs of the six indicators. The lowest rung of this outcome indicator (level 1) denotes “Behavior Change,” such as “Observable or documented changes in classroom practices, hiring practices, or student engagement” (data not shown). The next lowest rung of this outcome indicator (level 2) denotes “Awareness Growth,” such as “Pre/post assessments, survey data, or testimonials showing increased understanding of DEI concepts among colleagues or students.” The latter two strategies, or 40% (2/5), reside in the level 3 indicator, “Cultural Shifts,” such as “Departmental or programmatic norms evolving.” Eleven percent (*n* = 2/18) of the awardee strategies are classified across two unique TAE outcomes, with one in both “Inclusive Leadership and Representation” and “Impact on Attitudes, Awareness, and Behavior” and the other in both “Inclusive Leadership and Representation” and “Engagement in Institutional Learning and Transformation” (data not shown). See these 18 TAE strategies coded into four categories of impact, lettered A-D (Supplementary Table 3).

### 3.3 Distinguishing characteristics of the awardees

To identify salient features of the awardees, we used the modified grounded theory approach, explained in “Materials and methods” to analyze themes that emerged across conversations. Awardees’ experiences and perspectives on their teaching coalesced in three themes: (1) Adoption of transformative teaching approaches; (2) Teaching as a calling; and (3) Advancing changes in teaching and learning beyond the classroom. These themes contextualize the awardee strategy findings described in Tables 4, 5.

#### 3.3.1 Adoption of transformative teaching approaches

Awardees described three subcategories of activity: (a) creating participatory learning environments that engage all students, (b) implementing evidence-based active learning and teaching practices, and (c) offering experiential, practice-oriented learning to promote career readiness.

All 12 awardees described efforts to create learning environments that promote the acquisition of knowledge, skills, or attitudes described in the course syllabi or program learning objectives. Each also noted they stretched their teaching to build human connections for ensuring inclusion in whole-class learning. They use strategies that convey that all students belong and to “…make sure that the voices that are less heard are heard.” While maintaining their role as both content and methods experts, or sages, the awardees serve as more as learning guides (46) who aim to engage all students in their own learning and in co-growing peer learning. The awardees center their students’ learning experience by fostering intentional, authentic, and equitable connections with students and by seeking out quiet, struggling, and checked-out students for inclusion. One awardee shared, “[S]ome of my best students are the students who would have flunked out if I didn’t say: ‘Hey, I can tell something’s going on. I don’t need to know. But let’s figure out how you move forward.’” Awardees also promote the value of student peers as fellow student learning guides, asserting that “education happens when people talk to each other.” Awardees view these strategies as important contributors to student success.

To achieve successful learning environments, awardees intentionally incorporate multiple evidence-based teaching strategies into their courses. They mentioned a variety of scaffolded and interlaced skill-building approaches, including writing-intensive modules, flipped classroom techniques, critical questioning (with some using Socratic methods), peer mentoring, and innovative feedback. One awardee shared, “I’ve leaned in hard on the writing-intensive coursework and trying to figure out how to teach students to make clear and concise arguments for populations and to lead with data.” They also engage students in discussions by stimulating conversations with cognitive dissonance and other approaches. For example, an awardee explained, “One of my approaches is a dialectic emphasis in the in-class discussion, moving students away from black-and-white thinking to gray-area thinking.” These strategies are used to spark active learning, prepare students with critical thinking skills, and foster engagement and mutual learning. For example, an awardee said that they, “…encourage others to respond so the discussion becomes multi-directional and, as a group, we come to a shared understanding of concepts and issues.”

Awardees often design learning activities that are experiential and incorporate community-engaged practice. For example, one awardee established an annual study abroad program where students can learn to, “…recognize and value the knowledge and experience of community members and country partners in their research.” These practice-based pedagogical approaches set up students, after graduation, for public health practice and increase their possibility of impact (47, 48). One awardee explained that in their class, “students do real-world activities that they can then translate to career readiness.” Another awardee shared, “[I]f students can apply what they’re learning in the classroom to make a real-world impact…[it] is ultimately going to be the most effective way to teach.”

#### 3.3.2 Teaching as a calling

In the theme of teaching as a calling, many awardees consistently described their passion for teaching and student learning, along with a commitment to lifelong learning and reflective practice. For these awardees, teaching involves continual learning.

Most of these awardees share a deep sense of calling along with conviction and responsibility to their teaching vocation. One noted that, “It is the gift of my life to be able to do this work, and I revere it in that way.” Another awardee said, “a lot of faculty, especially early in their career, don’t realize that one of the greatest joys of being a faculty member is teaching.” Awardees’ commitment to teaching is amplified by their own value for public health. They also are aware of their contributions to their students’ future roles in protecting the health of the public within their own careers. Many describe their teaching as a fulfilling way to connect with students during such an important stage in their life journey. An awardee who teaches introductory-level classes shared that it is, “…such a joy because I can catch students from the very first semester and focus on those skill sets that will help them be successful, not only in that semester, but in subsequent ones.”

The awardees model lifelong learning and curiosity, along with humility, for their students and colleagues. They commonly described a practice of reflection to help them continually grow “and evolve over time” as teachers committed to creating transformative classrooms. One awardee shared “We have to spend some time looking back on what worked and didn’t work, what felt good, and what felt bad, [and] ask [hard questions]—we’ve got to get brave.” Another awardee mentioned, “The thing that has been very helpful to me is allowing myself to be humbled by what I don’t know, thinking of every year as a quest to learn more about the subject I teach, and to let [teaching] be a learning experience.” These awardees establish habits of exploring new ideas and incorporating new methods into their instruction—noting topics as varied as inclusive and anti-racist critiques and practices, artificial intelligence (AI), arts integration, linguistics, simulation, and, of course, teaching pedagogies—because, as one awardee stated, “Making changes along the way…is essential for my teaching.”

#### 3.3.3 Advancing changes in teaching and learning beyond the classroom

**Figure 3:**
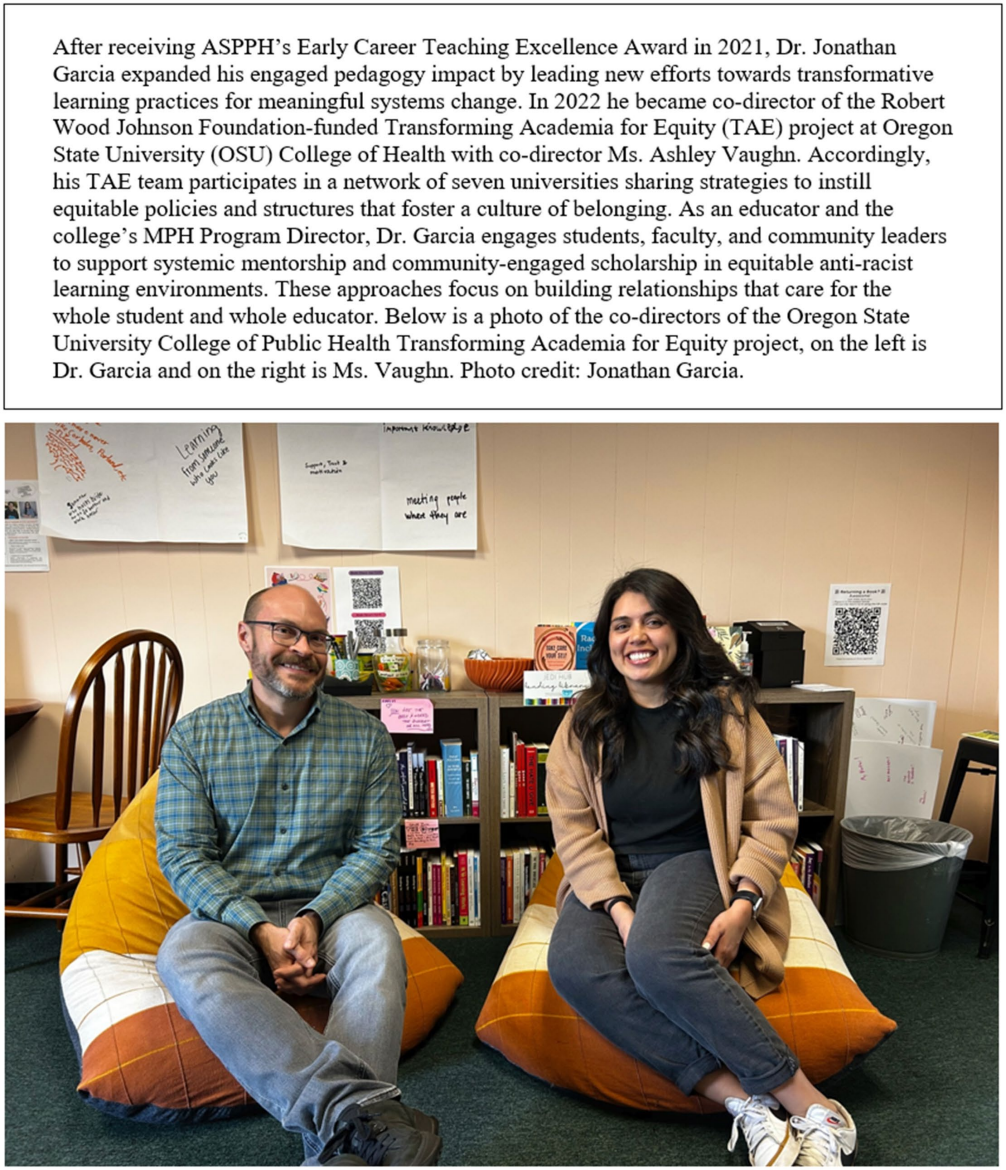
The single early career teaching excellence awardee who is also a TAE grantee.

Some awardees described their efforts in cultivating learning and improving education as a collective effort involving students, faculty, staff, community members, and institutional leaders at levels beyond their own classrooms—across departments, programs, and university systems. They work to shape the future of their institutions through leading accreditation processes, revamping MPH and DrPH curricula, mentoring other instructors, and promoting evidence-based teaching and learning. For example, one awardee shared that they are, “…involved more in the curriculum and the rollout of teaching in the department, pushing forward…a department-wide teaching philosophy of collaboration.” Another stated they volunteer in their university teaching center in “teaching and mentorship of faculty all across [the institution].” One awardee is a co-PI for his school’s RWJF-funded Transforming Academia for Equity (TAE) project (Figure 3). He along with other administrative leaders noted using their role, institutional platforms, and/or recognition (e.g., their award, directorships) to model and advocate for overall systemic change. A specific example of department-wide change was led by another awardee who implemented, evaluated, and promoted a successful DEI reflection tool. A demonstration of systemic change was noted in another awardee’s role with their team in piloting, evaluating, and operationalizing a successful peer advising model across the school.

#### 3.3.4 Institutional facilitators and barriers to teaching excellence

Awardees conveyed institutional-level facilitators and barriers to expanding innovative pedagogy in their educational and teaching-related community-engaged practice settings. These facilitators and barriers also contextualize the awardee strategies “Deploy strategic systemic levers of change” (Table 4) and “Execute institutional change strategies” (Table 5).

Two facilitators emerged in the study. The top enabler for awardees’ own effective teaching was represented by senior leaders who encourage, mentor, and nurture them along their career pathways. One awardee said, “Buy-in and the support from leadership, from my chair, and from the dean’s office again has been so incredibly important.” The second enabler was teaching-related learning communities where awardees and teaching colleagues can “come together and learn about teaching, learn about our students” as well as share ideas and challenges. An awardee described these collaborative teaching spaces as “very helpful, morale boosting, encouraging, and just professionally healthy.”

Awardees discussed five barriers in which university culture and structures experienced in academic public health, particularly at research-focused institutions, can hinder innovative pedagogy. First, several awardees noted that research-focused systems reward traditional research at the expense of teaching. One awardee observed, “Teaching is diminished in an environment like ours, which is soft-money oriented and where the cachet and the definition of success almost entirely revolves around the research funding you bring in.” Second, awardees discussed how the over-emphasis on student course evaluations to determine quality of teaching can stifle creativity in the classroom and, consequently, diminish both pedagogical innovation and student learning. An awardee shared, “If we were doing a better job of actually evaluating teaching, that would free us to make students angry…when evaluation on teaching is a hundred percent dependent on the students saying, ‘I like her,’ that’s a barrier.” Some awardees communicated the value of incorporating alternative evaluation methods, such as faculty peer observations. Third, awardees described a lack of time for pedagogical innovation, professional development in teaching, and scholarly teaching, pedagogical research, and related writing and publishing. One awardee said, “I need both the time to manage and grow the program and teach everybody, and then also to educate myself.” Fourth, awardees recognized opportunity gaps in support and systems for quality teaching, in particular systematic mentorship to advance their effectiveness as teachers. An awardee noted “We definitely have mentorship opportunities at my school, but I wish they were more institutionalized.” Fifth, the shifting landscape on diversity, equity, inclusion, and social justice (DEIJ) challenges present barriers to advancing excellence in teaching. Awardees at such institutions noted the strain it puts on sustaining collaborative teaching and learning efforts with communities, and that it is “really hard to keep it going long-term.”

## 4 Discussion

This study describes academic public health teaching strategies used in implementing FTF 2030 recommendations and aligning with TAE rubric’s desired outcomes. This study also elucidates the distinguishing characteristics of 12 ASPPH Early Career Teaching Excellence Award winners (“awardees”) who serve as key change agents in innovating teaching. Four findings emerged, that awardees: (1) implement strategies aligned with all three FTF 2030 objectives and all three TAE desired outcomes; (2) are educators who: emphasize student-centered teaching; model human connections with students for mutual learning both within the classroom and in engaging with communities; and, contribute to institutional change through curricular and faculty development; (3) view teaching as a vocation marked by joy in influencing student learning and exemplified by a passion for continual learning and reflective practice to teach well; and (4) thrive with *support* from leaders and participation in teaching and learning communities but encounter *barriers* such as the low value placed on teaching versus research-focused institutional priorities and overuse of student evaluations to judge educational quality. These findings offer insights for educators and guidance for institutional leaders to promote pedagogical innovation and opportunity, engagement, and excellence in education for public health.

### 4.1 Awardees’ implementation of FTF 2030 recommendations and TAE desired outcomes rubric

Awardees implemented teaching strategies aligned with all three FTF 2030 objectives and all three desired outcomes in the TAE rubric. One reason awardees may exemplify the aspirational goals of both frameworks is because the FTF 2030’s recommendations (Table 1) and TAE’s desired outcomes rubric (Supplementary Table 1) overlap with the award criteria (Figure 2). These overlaps include enhancing teaching excellence, practicing equity in teaching, promoting inclusive learning, teaching beyond the university, and exerting leadership in teaching and mentoring. For example, many awardees used self-critique to strengthen anti-racist strategies and integrate anti-racist pedagogy into course content, core FTF 2030 recommendations (Table 1) and TAE rubric (Supplementary Table 1). The awardees deepened learning and promoted student success through evidence-based practices, such as the use of case studies (49) and Transparency in Learning and Teaching (TILT) strategies. TILT strategies used by awardees aim to instill student “ownership of their education” (50), increase readiness for “real-life public health challenges” (51), and reduce gaps “especially first-generation, low-income, and underrepresented college students” (33).

We also hypothesize that the FTF 2030 and TAE recommendations capture the practices of exemplary educators in public health. The 50 FTF 2030 strategies coded into six categories of practice (Supplementary Table 2), and 18 TAE strategies coded into four categories of impact (Supplementary Table 3) implemented by the awardees support this statement. The TAE rubric sets a high bar because it requires measurable institutional-level impact. The preponderance of TAE strategies, 44% (8/18), fell under “Engagement in Institutional Learning and Transformation,” suggesting educators may more readily contribute to planning and learning efforts as compared with the other TAE outcomes. However, none of the awardees implemented the two highest levels of influence in the TAE “Impact on Attitudes, Awareness, and Behavior” outcome (Supplementary Table 1), with no evidence of reaching level 5 “Institutional Metrics” and level 6 “Data Sources.”

### 4.2 Awardees’ demonstration of transformative teaching and learning

We also found that awardees’ strategies sought to meaningfully include all students, mentor peers, and nurture community partnerships. To explain this result, we hypothesize that awardees are deeply committed to others—students, colleagues, and surrounding communities—and are driven by compassion, such as empathy and the desire to act (52). This “human-centric” competence (51) amplifies the awardees’ content expertise and technical skills. For example, by preparing students to practice with “empathy, humility, and compassion” (35), these learners are positioned for greater effectiveness in working within today’s pluralistic society. The increasing complexity of our modern human experience across diverse settings requires empathy as a skill in today’s workforce (53, 54). Further study may identify strategies to strengthen training in “human-centric” competencies for public health teaching faculty.

### 4.3 Awardees’ teaching vocation and investment in self, student, and institutional growth

Importantly, we found that most of the awardees described teaching as a vocation, marked by joy in contributing to student learning and a desire for their own continual learning and reflective practice to teach well. We hypothesize that teaching excellence is central to their mature professional identity, bringing fulfillment and motivation. Drawing from Parker Palmer’s “Let Your Life Speak,” these awardees are not merely pursuing personal goals in teaching but responding to a larger call (55). These mature leaders view learning as a lifelong process and always with an eye toward growth (56). Not surprisingly, FTF 2030 emphasizes that administrators, faculty, and staff are all *learners* who benefit from embracing growth mindsets and a plan for lifelong learning (33).

In *Positive Intelligence*, Shirzad Chamine highlights “Sage” over “Saboteur” mindsets, and depicts five Sage Powers — Empathize, Explore, Innovate, Navigate, and Activate — as important for fulfillment (57). Awardees who describe teaching as “the gift of my life” and experience “joy” in their profession reflect the Sage powers Chamine identifies. They connect deeply with students (Empathize), remain open to learning (Explore), and take action to improve outcomes (Activate). Such mindsets undergird ASPPH’s vision of a resilient educational system for public health (Figure 1), which is dependent on nimble teachers and their educational teams “constant transformation” (35) in effectively “serving in a rapidly changing and interconnected world” (Figure 1). Awardees, who are themselves resilient, are more likely to contribute to public health’s role in strengthening resilience at individual, community, *and* system levels (58).

Awardees continued to prioritize teaching as they advanced in rank (Table 3), assumed leadership roles, and took on additional responsibilities, contributing not only to personal fulfillment but also to institutional well-being and the public’s overall health (59). Furthermore, awardees embody Frenk’s “Education for Life” model, linking lifelong learning to health promotion and progressing along healthy and rewarding career paths in one’s own life (60) as important to their identity. Despite time limitations, funding challenges, and other professional pressures, these awardees remain focused on student success in preparing these future public health leaders. It is recommended that institutions consider incorporating teaching training systematically into their faculty onboarding and early career development planning and that they adopt systemic mentoring (61) to better prepare and support faculty in teaching effectiveness and for improving student learning experiences.

### 4.4 Facilitators and barriers to teaching excellence and strong student learning outcomes

Lastly, innovative pedagogy appears to thrive when awardees have *support* from leaders and the opportunity and incentives to participate in teaching and learning communities but is hindered by *barriers* such as the low value placed on teaching versus research-focused institutional priorities and the overuse of student evaluations to judge quality. We hypothesize that expanding innovative pedagogy depends on institutional support that affirms teaching as central to an educator’s professional identity and growth and simultaneously recognizes strong teaching as essential to academic public health’s mission. As one awardee described: “I do have staff now, which has been fantastic [and] helps to elevate what we’re doing and shows that our dean values [this work] …[H]aving that top-down support for teaching and learning is really essential.” Awardees linked their pedagogical innovation to facilitators such as leadership, mentoring, and recognition in promotion policies. A strong institutional culture for teaching excellence provides time, coaching, design support, and incentives for faculty development such as greater recognition in promotion policies (62). Without institutional and leadership buy-in, even highly motivated educators face challenges in sustaining innovation. Additional mentoring, training, and resources are recommended for institutions to support the development of faculty as effective educators. One practical solution is strengthening the weight teaching quality carries in the promotion and tenure process.

Course evaluations have been determined as flawed in measuring teaching quality, both because they have little to no measurable correlation with student learning and because there is a well-documented equity bias based on gender, race, and other marginalized factors (63–65). However, as mentioned by some awardees, course evaluations are consistently still used as a metric to evaluate teaching and student learning, failing to capture the complexity of effective education. Thus, we hypothesize that institutional cultures that rely on course evaluations create barriers to expanding innovative pedagogy.

Equity issues deserve attention along two lines: (1) weakened DEIJ commitments within academe and that extends to outreach with community partners and (2) nurturing teaching excellence among diverse faculty. Some awardees noted the scaling back of DEIJ efforts in recent years and its acceleration in 2025 has disrupted progress and has eroded trust among faculty, staff, students, and community partners. The effect within the classroom teaching has yet to be documented, thus representing an area of further study. Considering the nurturing of teaching excellence among diverse faculty, most awardees identified as non-Hispanic and White, reflecting broader faculty demographics and underscoring the need to expand public health faculty to include other races and ethnicities to advance inclusive excellence (66, 67). A final observation in this area is that most of the awardees identify as women (58%) drawing attention to the teaching labor that is more often borne by women (68).

### 4.5 Strengths and limitations of the study

#### 4.5.1 Strengths

Strengths include, first, its focus on educators in public health, a group often underexamined in pedagogical research. Second, this research generated the first analysis of FTF 2030 and TAE implementation practices of an exemplary educator cohort in public health. Third, it used an inductive approach that surfaced awardee-generated strategies. Fourth, the study incorporated a collective, reflective review process with awardees as coauthors, all but one of which was a study participant, in developing and submitting the manuscript.

#### 4.5.2 Limitations

Limitations include, first, partial participation in this sample (12 of 17 awardees, 71%). Second, since some of the awardees were interviewed many years after receiving their awards, with the first winner participating in this study 17 years later, they and some of the earlier awardees are less representative of an early career population now than at the time of their win (83% are currently 45 years and older). Third, there is an overrepresentation of schools versus programs with only one of the 17 winners (6%) hailing from a program. All winners are at schools, however, at the time of her award, one winner resided at Northwestern University Feinberg School of Medicine Program in Public Health. Fourth, there is no explicit reference to TAE in the interview protocol. Fifth, there is a dearth of evidence on award processes and systems (69–71), the design and utility of such award processes and systems (72), and their relationship with teaching effectiveness (73) that could help inform this study. Sixth, study participants were drawn from ASPPH’s Early Career Teaching Excellence Award winners’ pool leading to potential selection bias. For example, selection committees for each award are populated by prior award winners, who may prefer “like” candidates (74). Seventh, eligibility for the award is limited to ASPPH-member institutions (*n* = 155) as opposed to all schools and programs accredited by the Council on Education for Public Health (CEPH) (*n* = 256), the recognized accrediting body for academic public health (75). The ASPPH-member pool from which the awardees are drawn, which includes 61% of the CEPH-accredited schools and programs, may affect representativeness.

### 4.6 Implications

This study offers two broad implications. First, it proposes an aspirational educator framework for education in public health (Table 8) to guide educators and institutional leaders in expanding innovative pedagogy, inspired by this manuscript and related work (76, 77). The framework integrates the awardees’ FTF 2030- and TAE-aligned strategies into four levels. They follow: Level 1: Teaching as a core professional identity—in which teaching is tied to one’s vocation and purpose; Level 2: Teaching in the classroom—how educators animate their purpose through their pedagogy and teaching practice; Level 3: Teaching in the institution—collaborating to improve teaching across departments and schools, shaping curricula, programs, and policies; and, Level 4: Teaching beyond the institution—with communities at the local, national, or global levels.

**Table 8:** Aspirational educator framework for education in public health.

This aspirational framework integrates the awardees’ FTF 2030- and TAE-aligned strategies into four levels. The levels are affected by institutional facilitators and barriers identified in this study. **Level 1: Teaching as a core professional identity**. This level is about how deeply someone sees teaching as part of who they are and their professional purpose. It is shaped by personal reflection and how others (like leaders or peers) respond to their teaching. Qualities like empathy, curiosity, and creativity help strengthen this sense of purpose. **Level 2: Teaching in the classroom.** This level is about how educators bring their purpose to life with students. It includes building strong relationships, trying new teaching methods, and responding to what students need to prepare for working in and for public health. Joy and gratitude often come from seeing students grow and succeed. **Level 3: Teaching in the institution.** This level is about how educators bring their purpose to life with others at the institution to improve teaching and influence change in academic public health and other disciplines in support of the institution’s education mission. Educators collaborate with peers in shared efforts and collective action to help shape curricula, programs, practices, and policies. **Level 4: Teaching beyond the institution.** This level is about how educators bring their purpose to life with partners and communities outside the school—locally, nationally, or globally. Educators collaborate with these communities to co-create health and related solutions that benefit everyone. These efforts often reflect shared values and long-term trust. Institutional facilitators and barriers can strengthen or devalue an educator’s professional identity (Level 1) or accelerate or slow their strategies (Level 2–4).

Second, awardees emphasized the need for greater investments in institutionalized faculty support—including mentorship, protected time, and funding—to enhance teaching effectiveness. Those with formal leadership positions and access to resources were better positioned to influence systems and support colleagues and students.

Focusing on the inclusive excellence theme, ASPPH’s FTF 2030 Environmental Scan recommends for academic public health to “systematically and intentionally wrap around, coordinate, and integrate DEIJ and [anti-racist] efforts more across drivers and/or throughout multiple spaces and settings” (78). These wrap around activities correlate with the “container” approach noted in this study, again described as the intentional formation of a diverse, representative microcosm—spanning identities, hierarchies, and roles within academia and partner communities—to mobilize collective engagement for systemic change (45). This strategy was successfully implemented by one awardee, exemplifying a compelling case example of the single TAE grantee among the awardees whose strategies (Figure 3) reflect deeper institutional success in advancing DEI. With dedicated funding and staffing, the grantee and co-PI formed a guiding team to address systemic barriers to scholarly advancement. Their “container” approach brought together diverse stakeholders, from faculty and students to senior administrators and community leaders, creating a dynamic, cross-cutting structure. This approach figured prominently under awardees who “Execute institutional change strategies” (Table 7) and was the only approach to meet all six indicators in the TAE rubric’s “Inclusive Leadership and Representation” desired outcome (data not shown). It illustrates how intentional design, institutional support, and funding can produce results in equity-focused teaching and leadership.

### 4.7 Recommendations for future study

The research team identified several overall areas of recommendations for future study. The first is a collective review of major findings noted throughout the study, including awardees desiring more teacher training and a strengthening of educational evaluation practices to adopt multiple and holistic forms of evidence beyond the use of course evaluations. ASPPH’s institutional programs, practices, and policies report (62) provides several promising approaches for exploration. The second is to contrast the findings of this report with the perspectives of underrepresented minority educators in public health, especially those in non-tenure track positions (67, 79). As mentioned, most of the awardees are white (82%) and identify as a woman (58%). Only one recipient came from an ASPPH-member program (located within a medical school); the remaining 16 are from schools or colleges of public health. Future research across institution types, faculty title series, and demographics will help deepen understanding and inform efforts to strengthen academic public health’s impact across higher education.

## 5 Conclusion

In conclusion, awardee strategies depict faculty change agents who harness people, processes, and resources toward realizing ASPPH’s FTF 2030 vision of “health equity and well-being for everyone, everywhere through equitable, quality education for public health” (35) and who also help fulfill the TAE desired outcomes. Through transformational teaching practices, role-modeling, and leadership these educators influence students, peers, and local, domestic, and global community partners. They pursue these strategies even amid the lack of faculty development, mentorship, protected time, and resources, factors important to one’s scholarly career progression in teaching (76) and these educators’ ability to advance the academic public health educational mission. The awardees’ steady, often unrewarded efforts and progress reflect a deep commitment to student success, institutional excellence, and advancing public health.

This study identified two facilitators of teaching excellence, yet five persistent obstacles signal enduring challenges. In this pivotal era for academic public health (13), these barriers can be reframed as opportunities for innovation and advancement. Indeed, this time of flux and uncertainty invites a welcomed reimagining of academic public health. Educators and peers who aim to make a difference facilitating “student learning and personal growth” (19) are invited to “step into the breach, work through this uncomfortable and sometimes alienating time of change and engage in looking toward a healthier world with all those who work to promote and protect health” (35). Dynamic evidence-based teaching that prepares students for careers in public health offers a strong attraction for students to select such institutions (80). Moreover, we posit that community-engaged educators who align their pedagogy to community needs contribute to reimagining schools and programs of public health as credible and trustworthy bastions of teaching excellence.

Despite institutional cultures that prioritize research and marginalize teaching (81), these awardees exemplify just a small proportion of the transformative educators working across schools and programs of public health. All who teach are invited to reconsider their educational practices to inspire “a shared vision for a course, provide experience and a model, and challenge and encourage students with personalized attention and feedback” (19). This opportunity for self-examination is offered to all educators to enhance their effectiveness in co-creating learning (81) and cultivating students’ human, social, and professional potential for success in engaging with communities and supporting population health.

The deeply “human activity” of the “formation of a person” (82) by educators is highlighted throughout the findings. As an awardee noted “in public health, we’re not making widgets, we’re not on a factory line. We humans are the agents of change, so we have to do that hard work…and the change has to happen within the student” (a deidentified Early Career Teaching Excellence Award Winner, personal communication, June 2025). Another awardee similarly addresses the value of student agency and ownership in taking responsibility for their learning, noting “I have this limited time with [the students], either in the classroom or as a mentor, to plant all the seeds that I can plant, and then the student is the one that has to water the tree and bring it to fruition.”

Sullivan and Galea argue for a culture shift in what they see as an opportunity for “a creative rethink of how we value education, a spur to educational innovation, creativity, and research that could vault our institutions forward through a once-in-a-generation improvement” (83). Allen advises taking advantage of the current “chance to course-correct” in higher education and suggests a “framework of confident pluralism—inclusion and belonging, academic freedom and mutual respect” (28) as a path forward for academicians. Likewise, Chávez and Turalba (2006) advocate for a “pedagogy of collegiality” in public health in which “[t]eachers affect the social change process” by “politically savvy faculty able to train MPH students in ‘real-world’ applications of community-based participatory approaches” (84). Such innovative and inclusive teaching benefits institutions by increasing opportunities to retain students and prepare them to graduate (85) with the essential knowledge, skills, and attitudes for career success that employers are seeking (86). As FTF 2030 notes, we all are learners *and* drivers of change—university leaders, faculty, staff, students, and partners—and thus all are called to contribute to transforming education for public health into the future.

## Data Availability

The datasets presented in this article are not readily available because per the “Confidentiality Rules and Procedures” in the “Terms of Participation” agreement that all study participants (a.k.a. “awardees”) signed, “All data are kept confidential and will not be shared with anyone except the internal ASPPH evaluation team.” Requests to access the datasets should be directed to eweist@aspph.org.
