# Supplementary material for: Strategies and distinguishing characteristics of faculty change agents teaching public health: a study on innovative teaching in higher education
Source: Front Public Health. 2026 Mar 18;14:1694800. doi: 10.3389/fpubh.2026.1694800 (PMC13038930; doi:10.3389/fpubh.2026.1694800)
Supplement: Supplementary file 1 [file Table_1.docx]

## Transforming Academia for Equity (TAE) Desired Outcomes Rubric

## for the ASPPH Early Career Teaching Excellence Award Study (May 2025)

**Inclusive Leadership & Representation (IL&R)**

**Definition:** Demonstrates a strong commitment to amplifying underrepresented voices and advancing equity through leadership roles, initiatives, or collaborations that influence institutional change.

| **1. Leadership Roles** | **2. Initiatives Led** | **3. Collaborations** | **4. Recognition** | **5. Demographics of Beneficiaries** | **6. Data Sources** |
| --- | --- | --- | --- | --- | --- |
| DEI-focused committees, faculty workshops | Programs that support marginalized groups, mentoring programs | Cross-departmental partnerships with equity-focused goals | Awards, funding, acknowledgement | Representation and reach | CV, letters of support, reports |

**Sample Measurable Indicators:**

**Engagement in Institutional Learning & Transformation (EIL&T)**

**Definition:** Leads or substantially contributes to institutional learning and planning efforts related to DEI (i.e. workshops, assessments, curriculum redesign, policy development).

**Sample Measurable Indicators:**

| **1. Workshops/Trainings** | **2. Curriculum Work** | **3. Policy Influence** | **4. Assessment Tools** | **5. Institutional Planning** | **6. Data Sources** |
| --- | --- | --- | --- | --- | --- |
| Number of DEI sessions; participant feedback | Development/redesign of syllabi/courses to include inclusive content | Change admissions, hiring, promotion, classroom policies to improve equity | Creation or use of tools to evaluate DEI climate/outcomes | Contribution to DEI strategic planning, accreditation, mission/vision | Workshop rosters, feedback, policy meeting minutes, SP documents |

**Impact on Attitudes, Awareness, and Behavior (IAA&B)**

**Definition:** Demonstrates clear and measurable influence on peers, students, or institutional culture through shifts in attitudes, behaviors, or awareness related to DEI.

**Sample Measurable Indicators:**

| 1. **Behavior Change** | **2. Awareness Growth** | **3. Cultural Shifts** | **4. Peer or Student Testimonials** | **5. Institutional Metrics** | **6. Data Sources** |
| --- | --- | --- | --- | --- | --- |
| Observable or documented changes in classroom practices, hiring practices, or student engagement | Pre/post assessments, survey data, or testimonials showing increased understanding of DEI concepts among colleagues or students | Departmental or programmatic norms evolving | Qualitative data describing how the work has shaped others' thinking or actions | Improved outcomes in equity-related KPIs (e.g., retention rates, faculty diversity, bias incident reporting) | Survey results, teaching evaluations, testimonials/interviews, comparative data |

**Citations:**

Magana L, Alexander L. Partnership with RWJF on Transforming Academia for Equity Initiative. Washington (DC): Association of Schools and Programs of Public Health; (2022). Internal document.

Espiritu R, Waetzig E, Watts S. Transforming Academia for Equity (TAE) Informational Webinar [Internet]. Princeton (NJ): Change Matrix; (2021 Nov 8) [cited N/A]. Available from: <https://anr.rwjf.org/templates/external/TAE_Webinarslides.pdf>
