# Supplementary material for: Strategies and distinguishing characteristics of faculty change agents teaching public health: a study on innovative teaching in higher education
Source: Front Public Health. 2026 Mar 18;14:1694800. doi: 10.3389/fpubh.2026.1694800 (PMC13038930; doi:10.3389/fpubh.2026.1694800)
Supplement: Supplementary file 2 [file Table_2.docx]

**50 FTF 2030 Strategies Coded into Six Categories of Practice**

1. **Prepare students for community-engaged practice (n=12)**
2. Designated leadership for community engagement that enhances community partnerships [TA-6; FCP-11]
3. Designated experiential education leadership to help manage and connect programs [FCP-11]
4. Practical community-oriented academic leadership [FCP-11]
5. Community partnerships with the department of health and cross-sectoral collaboration with K-12 [TA-6; FCP-10, 12]
6. Community partners to co-teach in an online DrPH program [TA-6; FCP-10, 12]
7. Infusing community partners into the classroom [FCP-10]
8. Capstone is aligned with local DOH accreditation [FCP-10, 12]
9. Creation of a new skill cluster around community-based participatory work [FCP-12]
10. Integrating students into rural communities in a scholars program [TA-6; FCP-10, 12]
11. Authentic learning in the classroom to prepare students for community work [FCP-12; TA-8]
12. Student engagement in practice-focused, real-world activities to prepare for entering the workforce FCP-12; TA-8]
13. Creation of a community of practice among school and non-school partners [FCP-10; TA-6]
14. **Use student-centered practices (n=11)**
15. Co-learning with students [IE-1]
16. Co-creating class norms as essential for student engagement [IE-1]
17. Pivoting to address critical issues of the day in responsiveness to the moment (which may require setting aside the lesson plan) [IE-1]
18. Supportive engagement of non-native English speakers in class discussions [IE-1]
19. Supportive engagement of quiet or unengaged students [IE-1]
20. Repeated classroom messages of belonging and acceptance [IE-1]
21. Equitable attention to all students interested in engaging in research [IE-1]
22. Attending to students' assets, especially when harmed by discrimination and bias [IE-1]
23. Attending to all students in an inclusive equitable manner, in particular those who are struggling [IE-1]
24. Cognitive dissonance strategies for students to see anti-racism more broadly [IE-1]
25. Building of a study abroad program to teach students another culture's lived experience and how to engage respectfully and equitably [IE-1; TA-6; FCP-12]
26. **Deploy strategic systemic levers of change (n=11)**
27. Designated leadership - Dean for DEI [IE-2]
28. Designated leadership - chairing a departmental DEI committee [IE-2]
29. Roll-out, evaluation, and dissemination of a DEI reflection tool [IE-1,5]
30. DEI course reflection tool that has leadership support and has led to documented curricular changes [IE-1, 2, 3, 4, 5]
31. Strategic plan to guide a culture of belonging and activities in and out of the classroom, including collaboration with other universities [IE-1, 2, 4; TA-6; FCP-10]
32. Preparing students to identify determinants of health for devising solutions to health inequities [IE-1; TA-7]
33. Evaluation of faculty engagement in a successful peer mentoring program that was adopted universally at the school [TA-9]
34. Guiding on reevaluating and improving peer teaching evaluation at the school [TA-9]
35. Support for improving teaching via teaching observations [TA-9]
36. Required pedagogy coursework for PhD students [TA-9]
37. Use of a container approach to implement systemic change (inclusive of community partners) [IE-1, 2, 3, 4, 5; FCP-10, 12]
38. **Apply pedagogical techniques (n=9)**
39. Self-critique to improve anti-racism teaching and learning [IE-1]
40. Shrinking the learning gap for marginalized students through high-impact learning practices [IE-1]
41. Intentional integration of anti-racist pedagogy in the classroom [IE-1]
42. Use of high-impact practices [TA-8]
43. Use of case-studies [TA-8]
44. Transparency in Learning and Teaching (TILT) strategies [IE-1]
45. Teaching students equitable research methods [IE-1; FCP-12]
46. Training students and others to become better researchers [TA-9]
47. Pushing to move beyond inclusion to bravery in guiding students to step out of comfort zones [IE-1]

**E. Lead in faculty development (n=4)**

1. Delivery of teaching talks [TA-9]
2. Volunteer counseling work at the university's teaching center [TA-9]
3. Outreach to others to elevate their teaching and assessments [TA-9]
4. Creation of a faculty orientation workshop that includes teaching strategies [TA-9]

**F. Implement extracurricular strategies (n=3)**

1. Leading with racism and anti-racism exploration as an initial fundamental step of understanding systems of oppression [IE-1]
2. Creation of a listening session with marginalized students [IE-1]
3. Student coordination and implementation of a speakers' series [TA-8]
