# Supplementary material for: Strategies and distinguishing characteristics of faculty change agents teaching public health: a study on innovative teaching in higher education
Source: Front Public Health. 2026 Mar 18;14:1694800. doi: 10.3389/fpubh.2026.1694800 (PMC13038930; doi:10.3389/fpubh.2026.1694800)
Supplement: Supplementary file 3 [file Table_3.docx]

**18 TAE Strategies Coded into Four Categories of Impact**

**A. Institutional Change Strategies (n=7)**

1. Development of a successful guiding team that used a container model in partnering with different aspects of the academic system, including the President and community partners, to implement change [IL&R-1, 2, 3, 4, 5, 6]
2. Co-creation of a departmental DEI committee [IL&R-1]
3. Activities with affinity groups to expand teaching beyond the classroom, make structural and institutional changes, engage within and across universities, and develop more inclusive teaching strategies [IL&R, 1, 2]
4. Changing of promotion and tenure guidelines [IAA&B-1, 3]
5. Leveraging the institution's investment in innovative teaching, curricula, and students’ sense of belonging within the college [IAA&B-3]
6. Creation and rollout of a DEI course reflection tool, though while not fully supported, has been evaluated and shared, along with an associated training that jumpstarted teaching conversations in the department [EIL&T-4, 6]
7. Implementation of a community of practice with an associated learning tool [IL&R-1, 2, 3, 5: EIL&T, 4]

**B. New Equity Content/Curricula (n=5)**

1. Creation of a new core curriculum for the undergraduate program [EIL&T-2, 3, 4, 5]
2. Redesign of the MPH to attract students interested in social justice [EIL&T-2]
3. Inclusion of a health equity focus in MPH online program [EIL&T-2]
4. Development of a Health Equity course which attends to ethical methods, data ownership with communities, and relationship building to avoid exploiting the community [EIL&T-2]
5. Creation of a study abroad program to expose students to diverse perspectives and others' lived experiences [EIL&T-2]

**C. Student-centered Equity-oriented Strategies (n=4)**

1. Student partnering to develop a culture of belonging through a justice, equity, diversity, and inclusion hub [IL&R, 1, 2]
2. Execution of a successful group advising model that incorporates peer mentoring for building connections among alienated students [IL&R-2; IAA&B-1, 2]
3. Holding of a listening session geared to students with marginalized identities [IL&R-2]
4. Use of student feedback to inform equity-based changes in the curriculum tied with evaluation and scholarship of teaching and learning [EIL&T-2, 4]

**D. Student Growth in Equity-oriented Learning (n=2)**

1. Measuring the impact of structural bias and cultural humility learning by students meeting the CEPH competencies [IAA&B-2]
2. Use of the Henrietta Lacks book to teach about research harm in communities, which has helped shift and improve students' thinking about working with communities [IAA&B-1, 2]
